# Removal of toxic heavy metals from river water samples using a porous silica surface modified with a new β-ketoenolic host

**DOI:** 10.3762/bjnano.10.25

**Published:** 2019-01-23

**Authors:** Said Tighadouini, Smaail Radi, Abderrahman Elidrissi, Khadija Haboubi, Maryse Bacquet, Stéphanie Degoutin, Mustapha Zaghrioui, Yann Garcia

**Affiliations:** 1 Laboratoire de Chimie Appliquée et Environnement (LCAE), Faculté des Sciences, Université Mohamed I, 60 000 Oujda, Morocco; 2 Centre de l’Oriental des Sciences et Technologies de l’Eau (COSTE), Université Med I, 60 000 Oujda, Morocco; 3 Group of material sciences, energy and environnement, ENSAH, Alhoceima, Morocco; 4 Unité Matériaux et Transformations UMR8207 (UMET), Equipe Ingénierie des Systèmes Polymères, Université des Sciences et Technologies de Lille, Bâtiment C6 salle 119-59655 Villeneuve d’Ascq, France; 5 Laboratoire GREMAN CNRS-UMR 7347 IUT de BLOIS, Université François-Rabelais de Tours, 15 Rue de la Chocolaterie, 41029 Blois, France; 6 Institute of Condensed Matter and Nanosciences, Université catholique de Louvain, Place Louis Pasteur 1, 1348 Louvain-la-Neuve, Belgium

**Keywords:** heavy metals, hybrid materials, β-ketoenol–pyridine–furan ligands, polluted media, porous silica, remediation

## Abstract

A new hybrid adsorbent material for the efficient removal of heavy metals from natural real water solutions (Moroccan river water samples) was prepared by the immobilization of a new conjugated β-ketoenol–pyridine–furan ligand onto a silica matrix. The thermodynamical properties including pH, adsorption isotherms, competitive adsorption, selectivity and regeneration were studied to investigate the effect of ketoenol–pyridine–furan–silica (SiNL) on the removal of Zn(II), Pb(II), Cd(II) and Cu(II) from aqueous solutions. An increase in adsorption as a function of pH and fast adsorption was reached within 25 min. The maximum sorption capacities for Zn(II), Pb(II), Cd(II) and Cu(II) were 96.17, 47.07, 48.30 and 32.15 mg·g^−1^, respectively. Furthermore, the material proved to be very stable – its adsorption capacity remained greater than 98% even after five cycles of adsorption/desorption. Compared to literature results, this material can be considered a high-performing remediation adsorbent for the extraction of Zn(II) from natural real water solution.

## Introduction

Nowadays, pollution by a large number of heavy metals in water sources is commonly observed due the constant economical growth of our modern society. This environmental issue is being seriously considered by different circles [1–2], given that heavy metal ions are known to cause health problems even at low concentrations in living systems [3–5]. Among these toxic metals included on the US Environmental Protection Agency's (EPA’s) list of priority pollutants, zinc, lead, cadmium and copper are considered as the most hazardous.

Solutions to remove heavy metals from polluted media have been thus proposed. These include several well-known analytical chemistry methods [6–16] as well as adsorption materials able to extract metal ions from aqueous solutions [17–23]. However, this latter solution presents numerous drawbacks [24–25].

A new generation of hybrid organic–inorganic silica adsorbents displaying superior properties have been recently proposed [26–33]. Our group has been active in this field with the preparation of a large set of chemically modified silica [34–36]. We could show, for instance, that their adsorption behavior is mainly dependent on the presence of donor atoms within the incorporated organic moieties [37–40].

In this context, β-ketoenol receptors, which are very important organic molecules, are also known for their potential to form stable coordination complexes with most transition metals [41–42]. The incorporation at the surface silica of a β-ketoenol group thus affords these hybrids the capacity to retain heavy metal ions. The group of C. Sanchez has prepared some mesoporous thin films functionalized with silylated β-ketoenol compounds as fast uranyl species sensors with high selectivity and sensitivity [43]. Our group has also recently prepared several β-ketoenols derivatives incorporated at the surface silica as effective and stable adsorbents for selective removal of toxic metals from water [44–48].

**Scheme 1:**
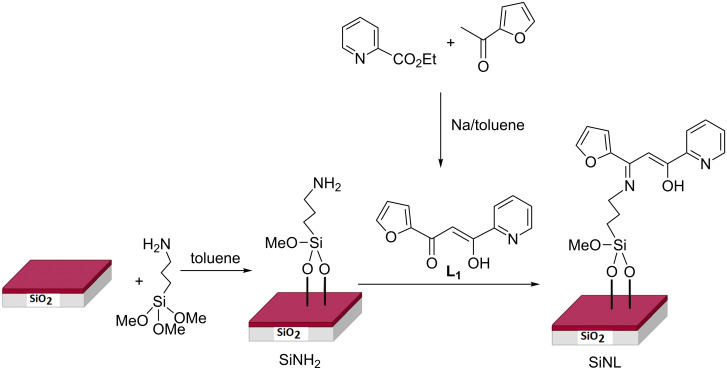
Synthetic route of our modified chelating material.

In the present work, we present the synthesis of a highly selective adsorbent material via covalent immobilization of a mixed ligand (β-ketoenol–pyridine–furan) onto silica particles (Scheme 1). The engineered system is low-cost, solid, chemically and mechanically stable as well as recyclable. It exhibited a high affinity and adsorption capacity for toxic heavy metal detection with less equilibrium time, a discovery that has significant environmental issues. Parameters that may improve the retention effectiveness of the metal ions have also been studied. The system was used for the confinement of lead, cadmium, zinc and copper metal ions from aqueous solutions as well as in natural water samples.

## Experimental

### Synthesis

Solvents and chemicals, used without further purification, were of analytical grade (Aldrich, 99.5% purity). Silica gel (particle size of 70–230 mesh, median pore diameter of 60 Å) (E. Merck) was activated before use by heating at 120 °C. The silylating agent (3-aminopropyltrimethoxysilane: Janssen Chimica) was used without purification.

(*Z*)-1-(Furan-2-yl)-3-hydroxy-3-(pyridin-2-yl)prop-2-en-1-one (**L_1_**). To a mixture of sodium (0.4 g, 17.39 mmol) and ethyl picolinate (2 g, 13.23 mmol) in 50 mL of toluene 1-(furan-2-yl)ethanone (1.91 g, 17.39 mmol) was added at 0 °C. The resulting solution was kept under stirring, at room temperature, for two days. The resulting precipitate was filtered, washed with the reaction solvent, dissolved in water and neutralized with acetic acid to pH 5. The organic layer, extracted with CH_2_Cl_2_, was dried and concentrated in vacuo. The resulting residue was chromatographed using silica and dichloromethane as eluent. Final product characteristic: yellow powder; yield: 30%; mp 102 °C; *R*_f_: 0.6 (CH_2_Cl_2_/MeOH 9:1)/silica. ^1^H NMR (DMSO-*d*_6_) 3.48 (s, 0.1H, keto, CH_2_); 7.29 (t, 1H, Fu-Hβ); 7.37 (s, 0.9H, enol, C–H); 7.42 (m, 1H, Fu-Hγ); 7.62 (m, 1H, Py-Hβ); 7.84 (t, 1H, Py-Hγ); 8.09 (d, 1H, Fu-Hα) 8.68 (d, 2H, Py-Hα). ^13^C NMR (DMSO-*d*_6_) 48.52 (1C, keto, CH_2_); 93.81 (1C, enol, C–H), 112.78 (1C, Fu-Cβ), 116.66 (1C, Fu-Hγ), 122.00 (1C, Py-Cδ), 126.32 (1C, Py-Cβ), 127.65 (1C, Py-Cγ), 137.14 (1C, Fu-Cα), 149.54 (1C,Py-Cα), 151.17 (1C, Py-Cε), 151.95 (1C, Fu-Cε), 178.71 (1C, C=O), 179.57 (1C; C–OH); IR (KBr, cm^−1^): ν (OH) = 3428; ν (C=O) = 1625; ν (enolic C=C) = 1515; MS *m*/*z:* 216 [M + H]^+^.

**3-Aminopropylsilica (SiNH_2_).** SiNH_2_ was prepared according to our published procedure [37].

**Pyridine-enol-imine-furan-substituted silica (SiNL).** After having refluxed 5 g of SiNH_2_ with **L_1_** in dry methanol (50 mL) for 24 h, the resulting hybrid was filtered, and Soxhlet was extracted with several organic solvents for 12 h and finally dried at 70 °C for 24 h. The material was characterized by elemental analysis, Fourier transform infrared spectroscopy (FTIR), scanning electron microscopy (SEM) images, thermogravimetric analysis (TGA) and nitrogen adsorption–desorption isotherms.

**Physical methods.** Atomic adsorption measurements were performed on a Varian A.A. 400 spectrophotometer. pH determinations were carried out with a pH 2006, J. P. Selecta s. a. pH meter. Microanalysis was performed at the Microanalysis Centre Service (CNRS). FTIR spectra were recorded on a Perkin Elmer System 2000 device. SEM imaging was run on a FEI-Quanta 200 microscope. TG/DTA were performed on a Perkin Elmer Diamond under a 90:10 oxygen/nitrogen atmosphere at 10 °C·min^−1^. The specific area was determined by using the BET equation. Nitrogen adsorption–desorption isotherm plots were obtained on a Thermoquest Sorpsomatic 1990 analyzer after the materials had been purged in a stream of dry nitrogen.

**Batch experiments.** The batch experiments were performed according to our published procedure [37]. Residual metal content was determined by atomic adsorption, using the following equations to determine the amount of adsorbed metal:

[1] $$Q_{\text{M}}=\left(C_{0}-C_{\text{e}}\right)\times V/W$$

[2] $$Q_{\text{W}}=Q_{\text{M}}\times M$$

where *Q*_M_ (mmol·g^−1^) and *Q*_W_ (mg·g^−1^) are the amount of the metal ion on the adsorbent. The aqueous solution volume, the weight of the adsorbent, the initial concentration of metal ion, the equilibrium metal ion concentration and the atomic weight for metals are designated by: *V* (L), *W* (g), *C*_0_ (mmol·L^−1^), *C*_e_ (mmol·L^−1^) and *M* (g·mol^−1^), respectively. The average data from duplicate analyzes were reported for each sample.

The performance of SiNL adsorption of Zn(II), Pb(II), Cd(II) and Cu(II) was carried out by stirring 10 mg of adsorbent with 10 mL of a solution of metal ions a concentration of 10–250 mg·L^−1^ at room temperature. The pH values were adjusted with dilute HCl and NaOH solution.

**Selectivity.** The selectivity sorption of Zn(II) ion was studied using solution containing: Zn(II), Pb(II), Cd(II) and Cu(II) ions (100 mg·L^−1^ of each, pH 6). Then, 10 mg of SiNL was added into 10 mL of the metal solution at room temperature for 60 min. The atomic adsorption measurements is used to measure the concentration of Zn(II), Pb(II), Cd(II) and Cu(II) before and after adsorption.

**Reusability.** Following the adsorption tests, 10 mg of SiNL was separated from the residue solution by filtration. The fractions containing unretained metal ions were examined by atomic absorption spectrometry. The sorbents were then rinsed once with acetonitrile (2 mL) and twice with distilled water (10 mL). The material was regenerated using 10 mL of HCl (6 M), and the reconditioned SiNL used in sequential experiments was used to repeat the adsorption procedure to achieve five cycles.

## Results and Discussion

### Materials and methods

The synthetic procedure of the adsorption material is given in Scheme 1. The first step refers to the synthesis of the target (*Z*)-1-(furan-2-yl)-3-hydroxy-3-(pyridin-2-yl)prop-2-en-1-one (**L_1_**) ligand in its stable conjugated enol tautomeric form. The reaction was carried out from ethyl pyridine-2-carboxylate and 2-acetylfuran via in situ Claisen condensation reaction [49]. The reaction of the activated silica gel with (3-aminopropyl)trimethoxysilane in toluene afforded amino groups onto the silica surface (SiNH_2_), which were then reacted with L_1_ under gentle conditions (reflux, 24 h) to form the newly chelating adsorbent SiNL (Scheme 1).

### Characterization of the adsorbent material

Elemental analysis was carried out for SiNH_2_ (C: 4.46% and N: 1.66%) showing that the amount of (3-aminopropyl)trimethoxysilane grafted on the surface of silica SiG is 0.92 mmol·g^−1^ whereas for SiNL (C: 6.54% and N: 1.71%) the amount of the ligand **L_1_** on the surface of SiNH_2_ is 0.14 mmol·g^−1^. The high carbon concentration in SiNH_2_ supports the anchoring of the silylating agent. The observed increase in both N and C content for SiNL indicates that the reaction with (*Z*)-1-(furan-2-yl)-3-hydroxy-3-(pyridin-2-yl)prop-2-en-1-one was successful.

**Figure 1:**
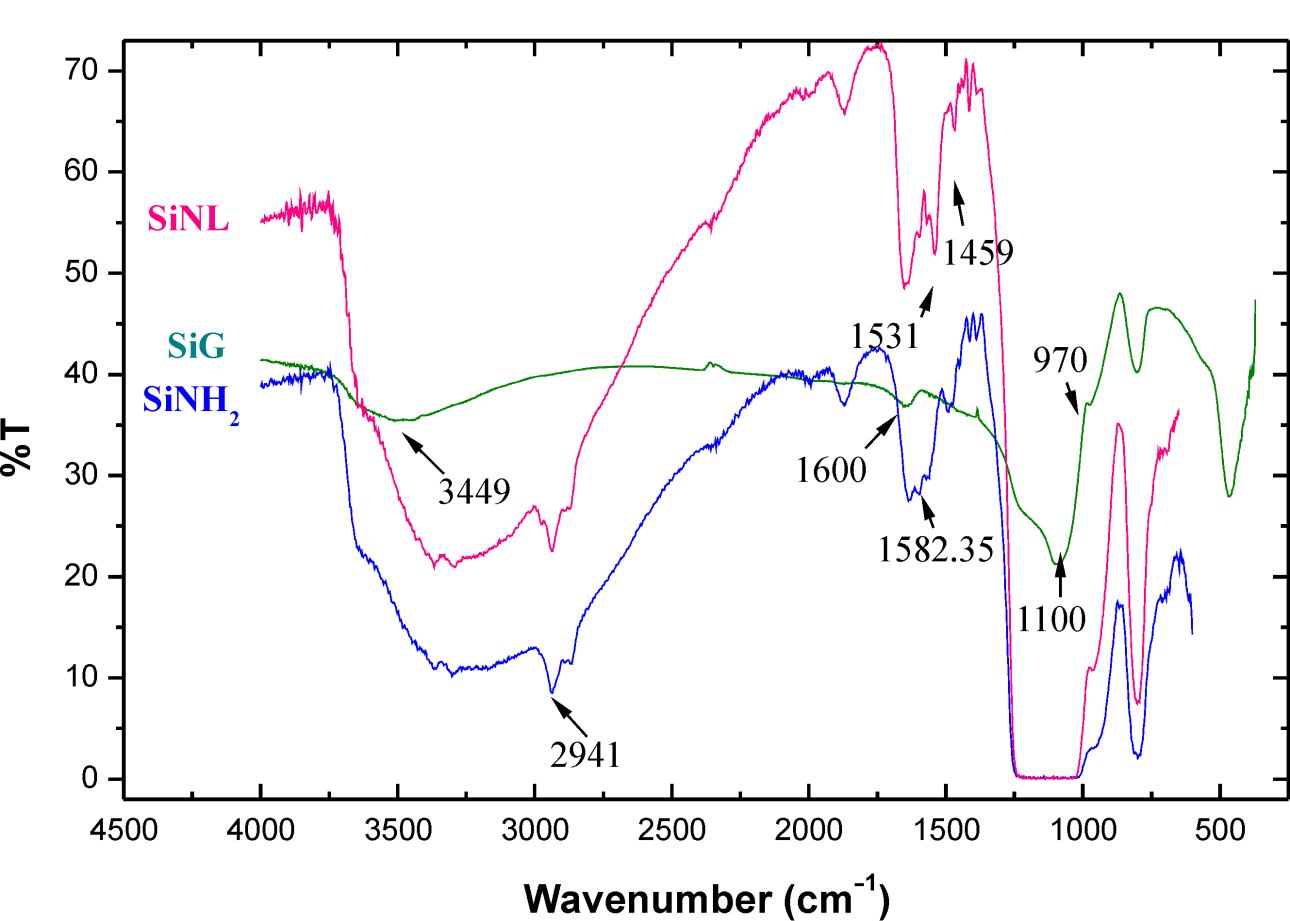
FTIR spectra of SiG, SiNH_2_ and SiNL.

**Figure 2:**
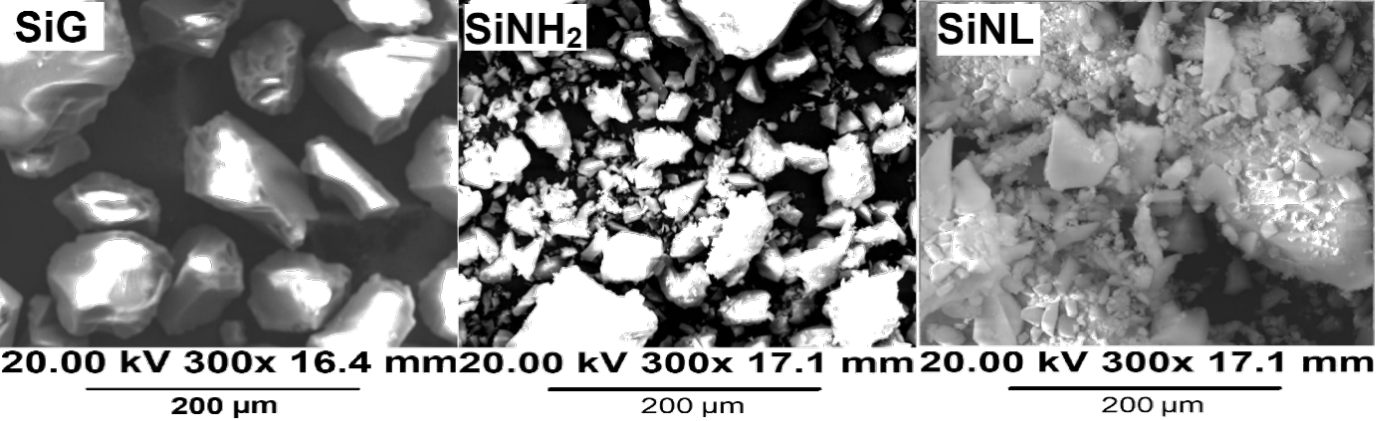
SEM images of free silica (SiG), SiNH_2_ and SiNL.

FTIR spectra of original silica gel (SiG), SiNH_2_ and SiNL are shown in Figure 1. The characteristics of the precursor materials (SiG, SiNH_2_) are consistent with literature [44–48]. In the SiNL spectrum, the stretching vibration of O–H band of material surface was obtained at 3351 cm^−1^ and the peak observed at 1050 cm^−1^ corresponds to Si–O–Si band, the strong bands observed at 2943 cm^−1^ are attributed to the stretching vibration of aliphatic C–H bands. The new ν(C=C) and ν(C=N) vibrations detected at 1459 cm^−1^ and 1531 cm^−1^, respectively, demonstrate the modification of SiNH_2_ with **L_1_**. Compared to the blank silica, the surface of the new material (SiNL) shown by SEM (Figure 2) became rough, which confirms the success of organic moieties filling the surface.

**Figure 3:**
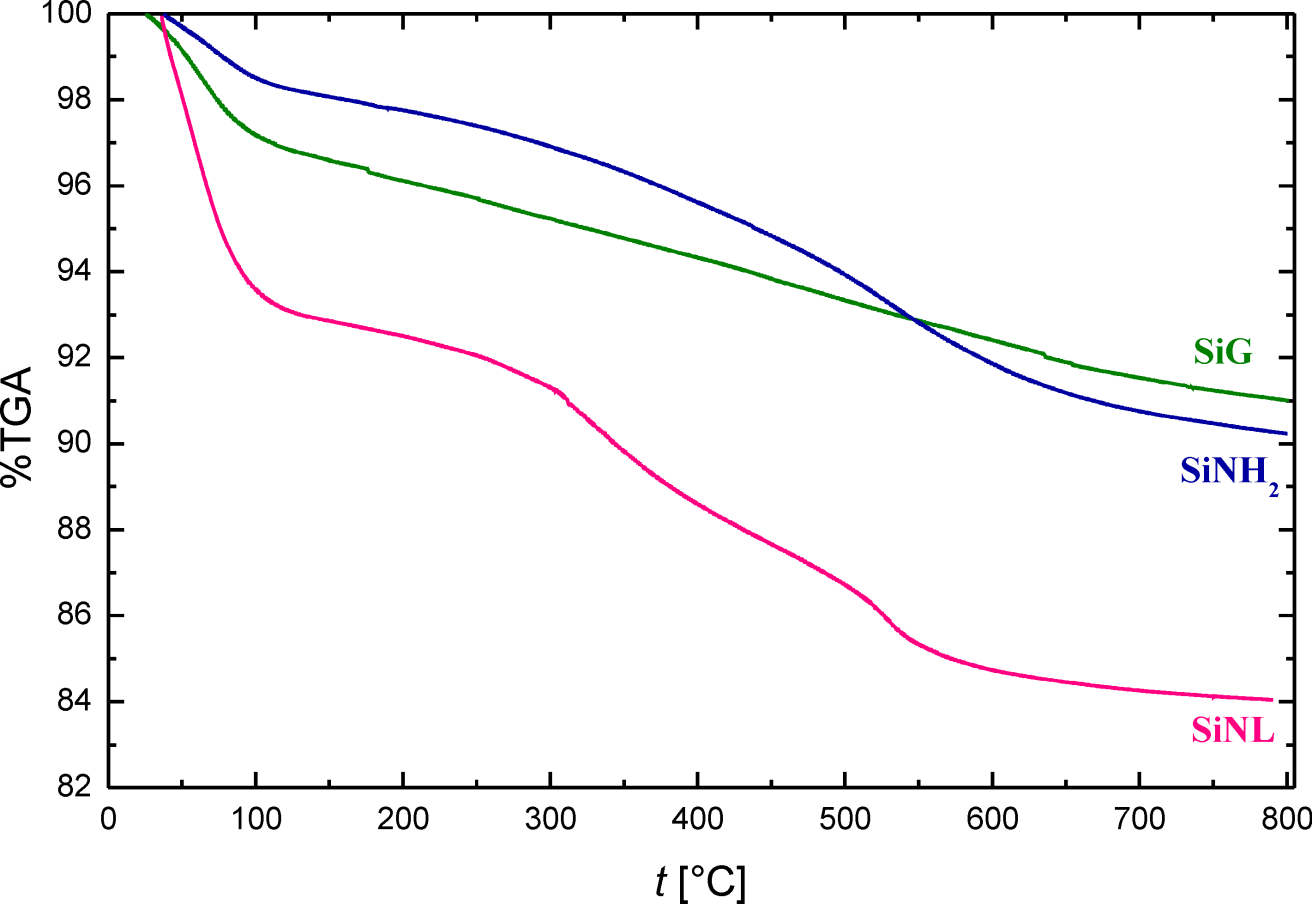
Thermogravimetric profiles of free silica SiG, SiNH_2_ and SiNL.

The thermal stability of SiG, SiNH_2_ and SiNL was evaluated by TGA (Figure 3). SiG presents a mass loss of 3.15% from 25 °C to 110 °C, which can be attributed to the release of water molecules [50]. A second mass loss of 5.85% from 110 °C up to 800 °C was detected, which presumably results from the release of silanol groups from the surface of the silica gel [48]. Similar to SiG, SiNH_2_ and SiNL also present two thermal steps. The first mass loss of 2.72% was assigned to the evaporation of adsorbed water. The second mass loss of 6.19% and 9.07% from 110 °C to 800 °C was attributed to SiNH_2_ and SiNL, respectively. This mass loss is attributed to the pyrolysis of the organic entities decorating the silica surface as well as to the condensation of the unreacted silanol groups [51–53]. These results prove that the SiNL system has been successfully fabricated and is thermally stable.

**Figure 4:**
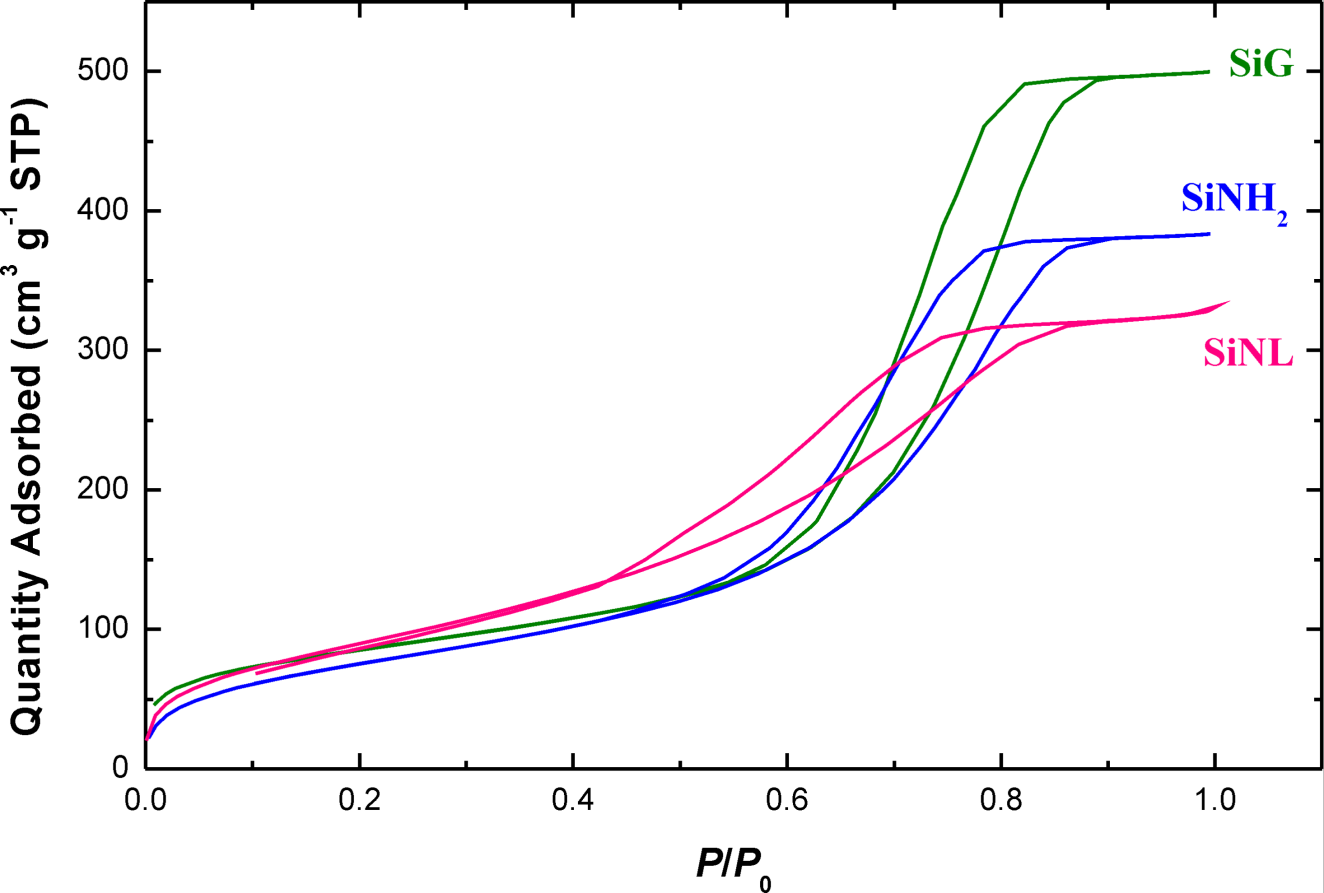
Nitrogen adsorption–desorption isotherm plots of SiG, SiNH_2_ and SiNL.

The specific surface area (*S*_BET_) of SiG, SiNH_2_ and SiNL was obtained by nitrogen adsorption at several pressures [54] and is shown in Figure 4. A clear decrease is observed after grafting. This is obviously due to the obstruction of N_2(g)_ access by organic moieties anchored onto the silica matrix, thus reducing its surface area [55].

The observed decrease of the initial specific surface area, *S*_BET_, from 305.21 ± 0.79 m^2^·g^−1^ to 283.08 ± 0.77 m^2^·g^−1^ and pore volume from 0.77 ± 0.002 cm^3^·g^−1^ to 0.69 ± 0.002 cm^3^·g^−1^ from free silica to SiNH_2_ results from the immobilization of organic moieties which can block the access of N_2(g)_ to the silica base. Further immobilization decreases the pore volume to 0.62 ± 0.01 cm^3^·g^−1^ for SiNL. The increase of *S*_BET_ to 339.84 ± 2.01 m^2^·g^−1^ for SiNL is presumably due to the increasing surface roughness, as noticed by SEM imaging (Figure 2), or due to the pore plugging of the support by the ligand.

### Solid–liquid adsorption of metal ions by SiNL

**Figure 5:**
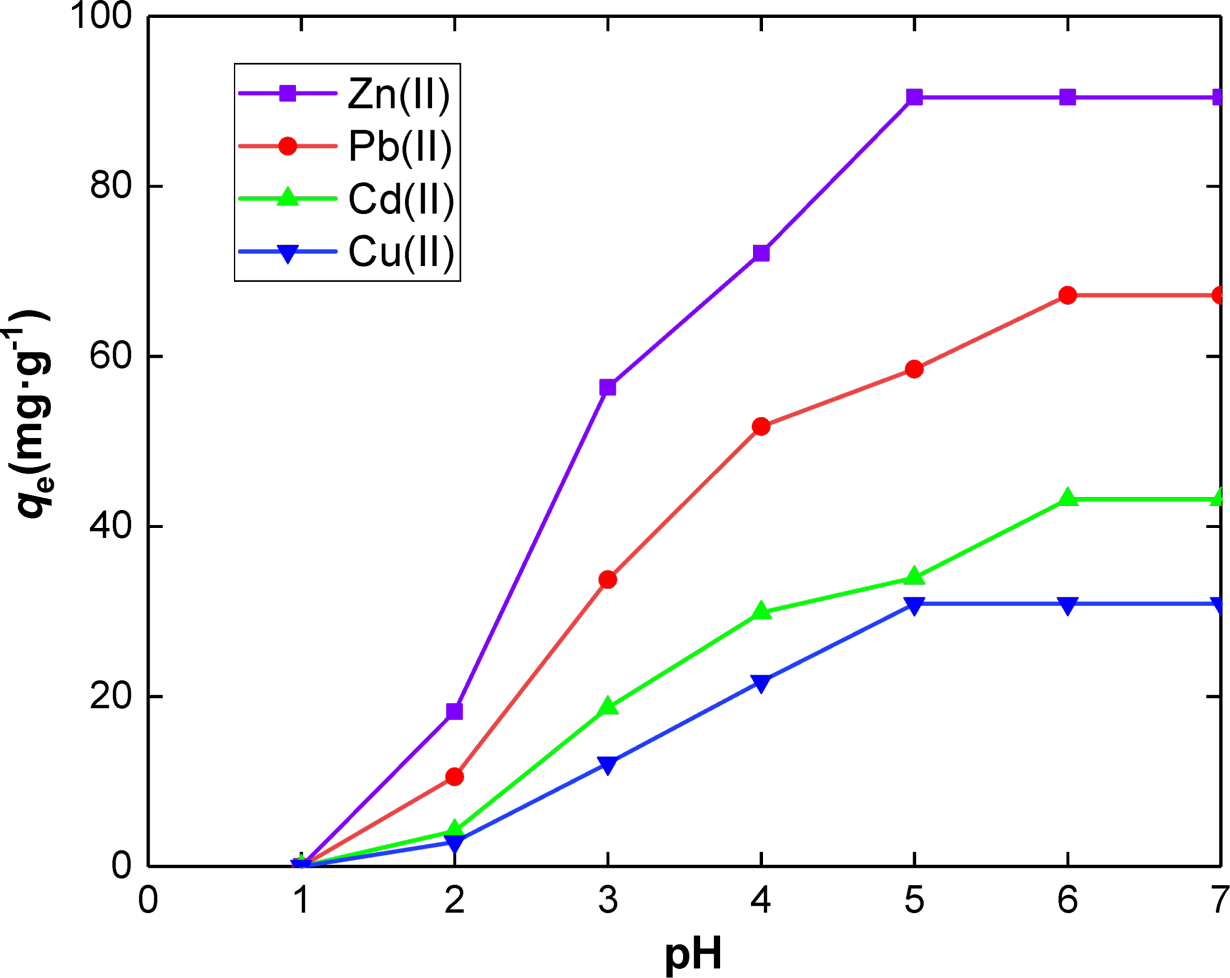
Effect of pH on the adsorption of metal ions on SiNL, Adsorption dose: *V* = 10 mL, *m* = 10 mg of SiNL at optimum concentration (100 ppm in each case), *t* = 35 min and 25 °C, ∆*q*_e_ = 0.3 (mg·g^−1^). (The optimum concentration means the initial concentration of metal ions required to reach a plateau shape).

**Effect of pH.** The speciation of metal ions in solution and the surface charge of the adsorbents can be influenced by the pH of a solution [56]. Donor groups attached to the adsorbents may be easily protonated or deprotonated to form different surface charges in solution at different pH values [40]. The effect of the pH on Zn(II), Pb(II), Cd(II) and Cu(II) sorption onto SiNL was investigated in the range of pH 1–7 using the batch method as shown in Figure 5.

The absorption of the metal ions increases with pH. When the pH is low, the retention of metal ions by SiNL is negligible. This is presumably due to the total protonation of the active chelation sites. As the pH rises, the protonation decreases, which tends to improve the chelation and therefore the adsorption of the metal ions. At pH > 8, the metal ion concentration decreases because of their hydrolysis. Actually, the adequate pH for the maximum adsorption of Zn(II) and Cu(II) was found at pH ≥ 5, and at 6 for Pb(II) and Cd(II). The best adsorption properties were identified for Zn(II) (Figure 5), which is presumably due to the higher stability of the formed Zn-ligand complex compared to other complexes formed with other metal ions. This can be indeed explained by the different binding affinity towards the adsorbent [57–58].

**Figure 6:**
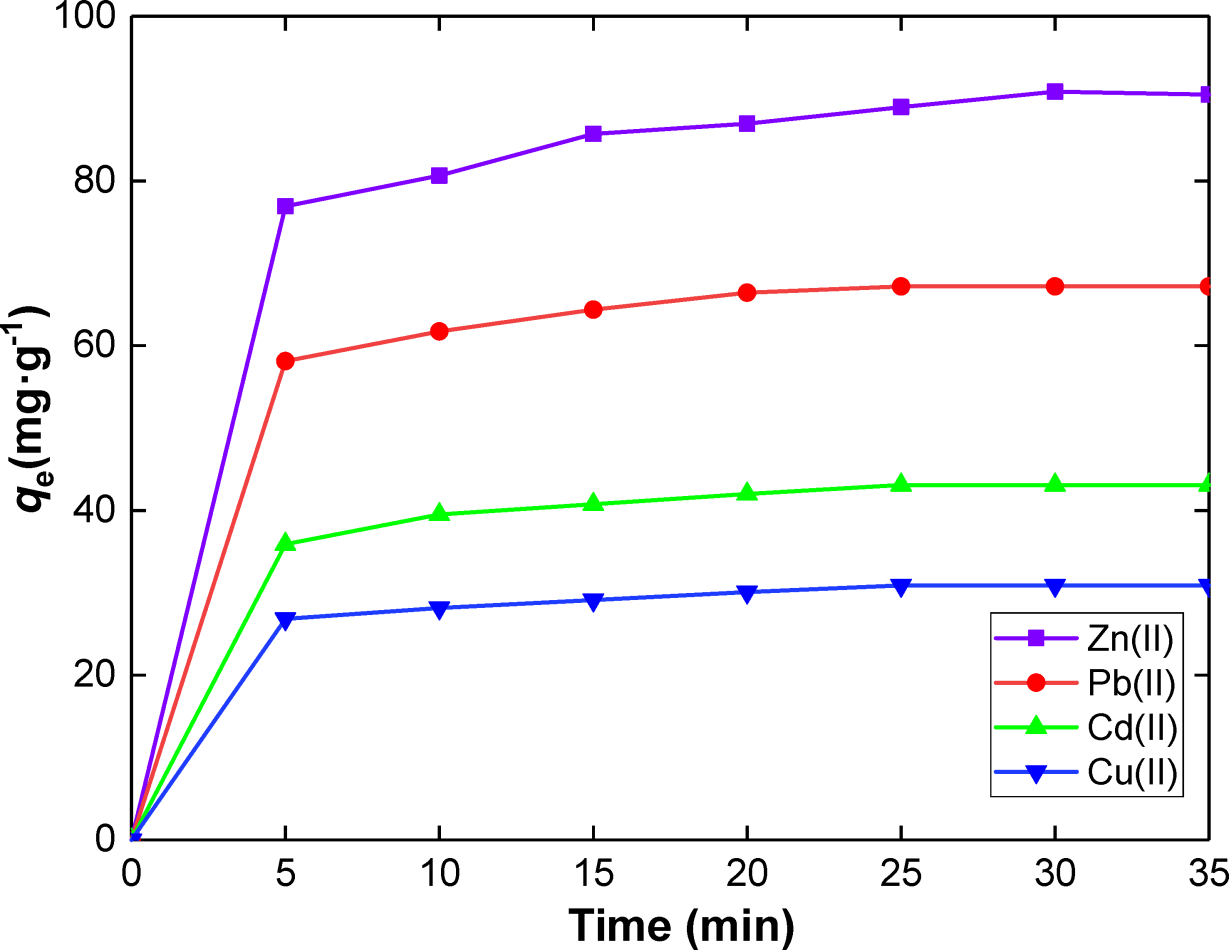
Effect of contact time on the adsorption capacity of Zn(II), Pb(II), Cd(II) and Cu(II) ions. Adsorption dose: *V* = 10 mL, *m* = 10 mg of SiNL at optimum concentration (100 ppm in each case), at pH 6 and 25 °C, ∆*q*_e_ = 0.3 (mg·g^−1^).

**Effect of contact time and adsorption kinetics.** The effect of contact time on the removal efficiency of Zn(II), Pb(II), Cd(II) and Cu(II) using SiNL was investigated (Figure 6). The adsorbed amount of metal increases sharply up to 5 min, after which a very gradual increase is observed to reach saturation after 25 min. The high amount of active sites of SiNL as well as the high solute gradient of concentration favors this behavior [59]. A contact time of 25 min was thus considered for all equilibrium adsorption studies.

First or second order kinetic models can be applied to evaluate the efficiency of the adsorption processes, and are therefore of the utmost importance to understand adsorption mechanisms [60]. The nonlinear equation for the pseudo-first-order model is recalled below:

[3] $$q_{\text{t}}=q_{\text{e}}\left[1-e^{-k_{1}t}\right]\;,$$

where *q*_e_ and *q*_t_ are the amounts of metal ions adsorbed on the adsorbent (mg·g^−1^) at equilibrium and at time *t*, respectively, and *k*_1_ is the rate constant of the first-order adsorption in min^−1^. The nonlinear equation for the pseudo-second-order can be written as follows:

[4] $$q_{\text{t}}=\left(k_{2}q_{\text{e}}^{2}t\right)/\left(1+k_{2}q_{\text{e}}t\right)\;,$$

where *k*_2_ (g·mg^−1^·min^−1^) is the pseudo-second-order adsorption rate constant.

**Figure 7:**
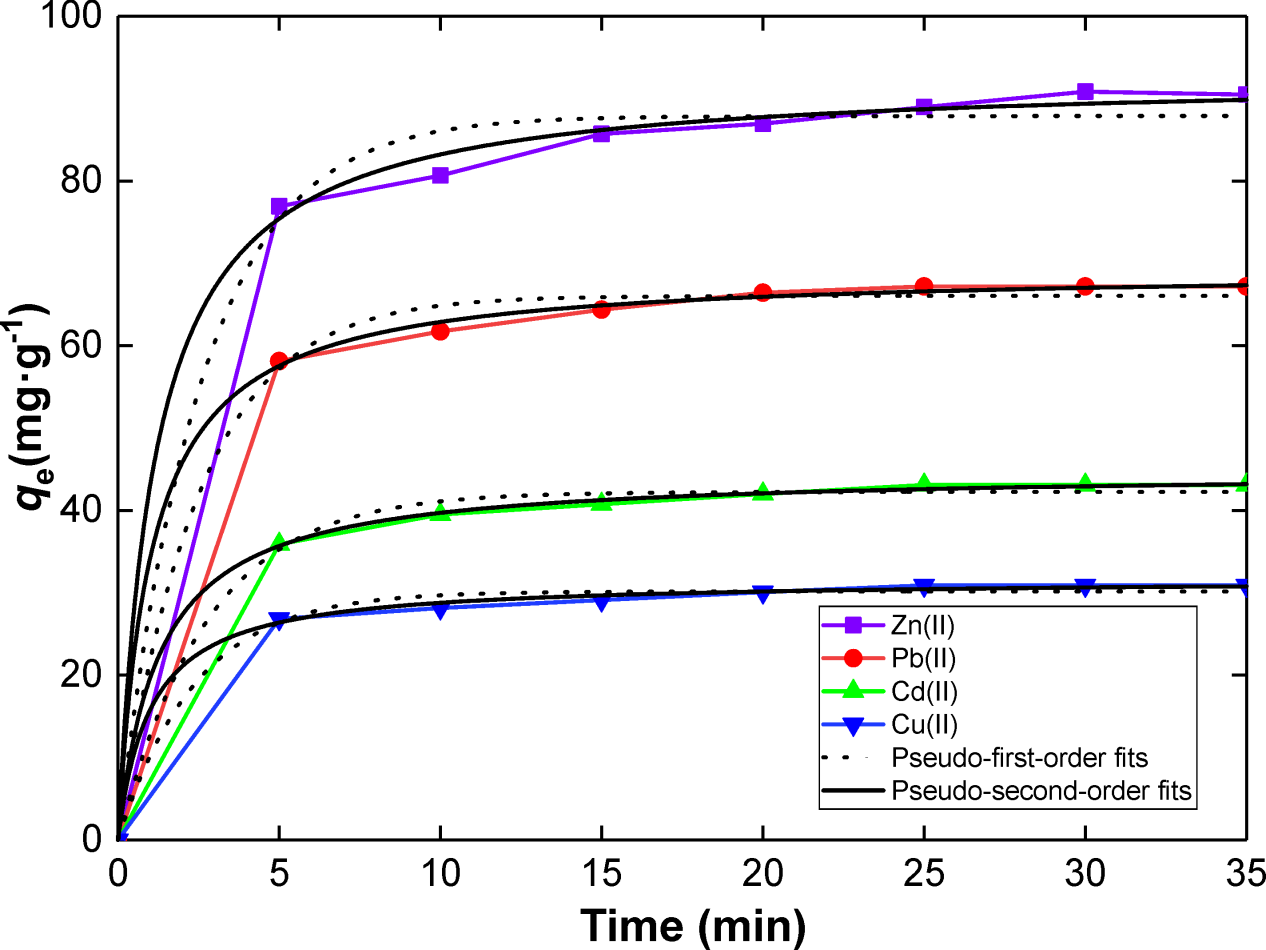
Pseudo-first-order and pseudo-second-order models fits for the adsorption of Zn(II), Pb(II), Cd(II) and Cu(II) ions by SiNL. Adsorption dose: *V* = 10 mL, *m* = 10 mg of SiNL using optimum pH (pH 6), optimum concentration (100 ppm in each case), and at 25 °C, ∆*q*_e_ = 0.3 (mg·g^−1^).

The kinetic rate constants for the adsorption of Zn(II), Pb(II), Cd(II) and Cu(II) by SiNL were determined by regression analysis (Figure 7). The data were best fitted by the pseudo second-order model (Table 1), which suggests that the adsorption of heavy metals in water proceeds by chemisorption [61–63], a phenomenon which could be attributed to the complexation reaction.

**Table 1:** Kinetics of heavy metal removal onto SiNL.

Parameters	Metal			
	Zn(II)	Pb(II)	Cd(II)	Cu(II)

*q*_e(exp)_ (mg/g)	90.48 ± 0.30	67.18 ± 0.30	43.10 ± 0.30	30.91 ± 0.30
**1st-order**				
*q*_e_ (mg/g)	87.87 ± 1.29	66.04 ± 0.72	42.25 ± 0.46	30.17 ± 0.39
*k*_1_ (min^−1^)	0.38 ± 0.05	0.40 ± 0.04	0.36 ± 0.03	0.41 ± 0.05
R^2^	0.991	0.995	0.995	0.993
**2nd-order**				
*q*_e_ (mg/g)	92.79 ± 1.05	69.30 ± 0.47	92.79 ± 1.05	92.79 ± 1.05
*k*_2_ (g/mg min)	(9.36 ± 1.3) × 10^−3^	(14.13 ± 1.3) × 10^−3^	(17.59 ± 1.1) × 10^−3^	(31.61 ± 4.5) × 10^−3^
R^2^	0.998	0.999	0.999	0.998

**Figure 8:**
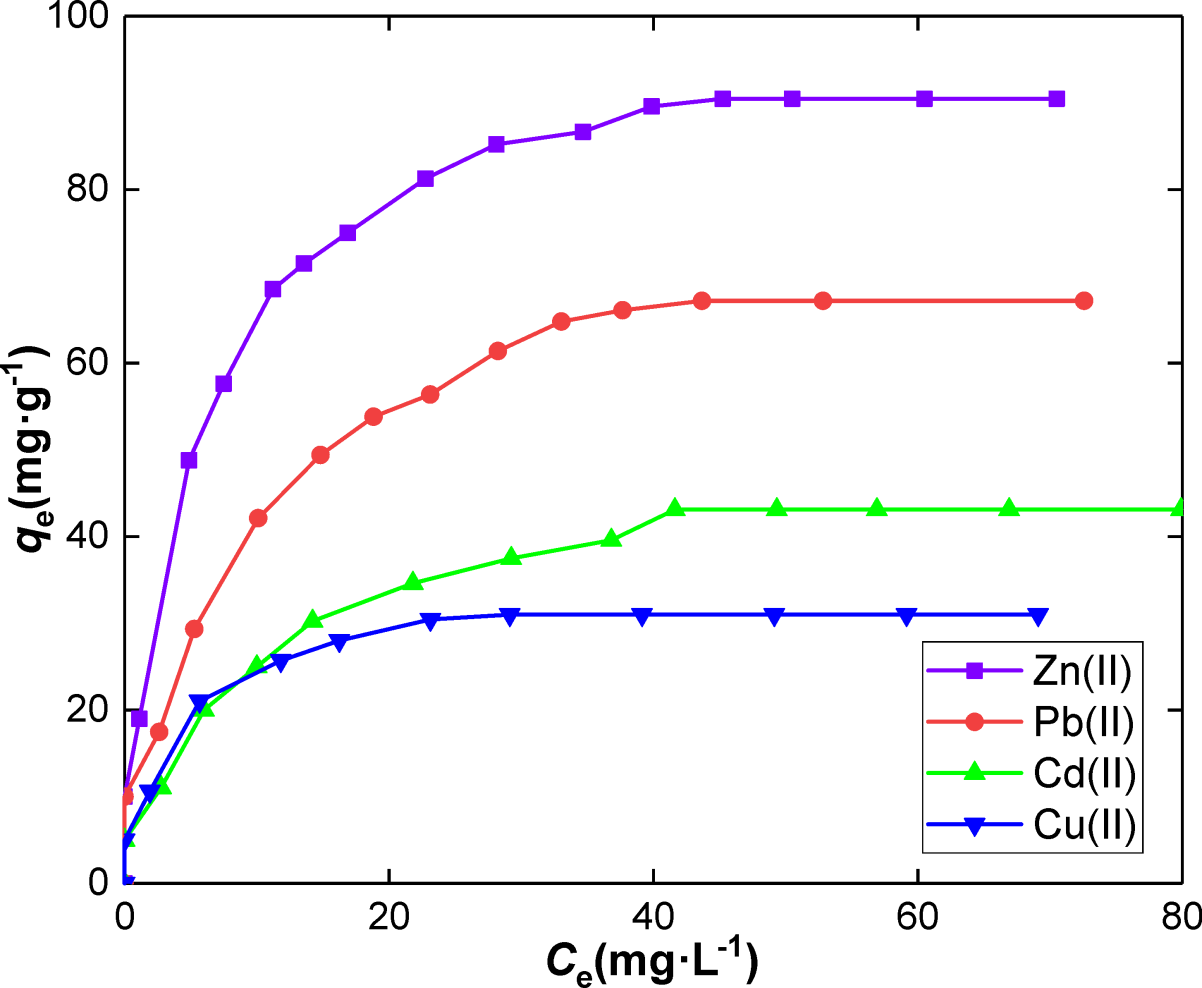
Effect of concentration on metal ion adsorption onto SiNL. Adsorption dose: 10 mg, *V* = 10 mL, at 25 °C and pH 6 for Zn(II), Pb(II), Cd(II) and Cu(II) ions, ∆*q*_e_ = 0.3 (mg·g^−1^).

**Effect of initial concentration in metal and isotherm modeling.** A detailed concentration study was conducted, and isotherm studies were carried out to interpret the metal retention by our system. Figure 8 highlights the increase in the retention capacity as a function of the increase in the initial concentration of Zn(II), Pb(II), Cd(II) and Cu(II) metals. The maximum adsorption was achieved gradually after an initial concentration of each metal of about 40 mg·L^−1^.

The adsorption isotherms allow the metal uptake per unit of adsorbent to be determined at equilibrium. The Langmuir isotherm model, which considers all adsorbent sites to be at equal energy with no adsorbent/adsorbate interactions, is one of the most popular.

The non-linearized form of the Langmuir isotherm equation is given below [64–65]:

[5] $$q_{\text{e}}=\left(qK_{\text{L}}C_{\text{e}}\right)/\left(1+K_{\text{L}}C_{\text{e}}\right)\;,$$

Where *q*_e_ is the amount of solute sorbed on the surface of the sorbent (mg·g^−1^), *C*_e_ is the equilibrium ion concentration in the solution (mg·L^−1^), *q* is the saturated adsorption capacity (mg·g^−1^) and *K*_L_ is the Langmuir adsorption constant (*L*·mg^−1^).

Another popular isotherm model is the Freundlich model which has been described by the multilayer sorption for the sorption on heterogeneous surfaces. The non-linearized Freundlich isotherm equation is recalled below [66]:

[6] $$q_{\text{e}}=K_{\text{F}}C_{\text{e}}^{1/n}\;,$$

where *q*_e_ is the adsorption capacity (mg·g^−1^), *C*_e_ is the equilibrium concentration of the solute (mg·L^−1^), *n* is Freundlich constant and *K*_F_ is the binding energy constant reflecting the affinity of the adsorbents to metal ions (mg·g^−1^).

**Figure 9:**
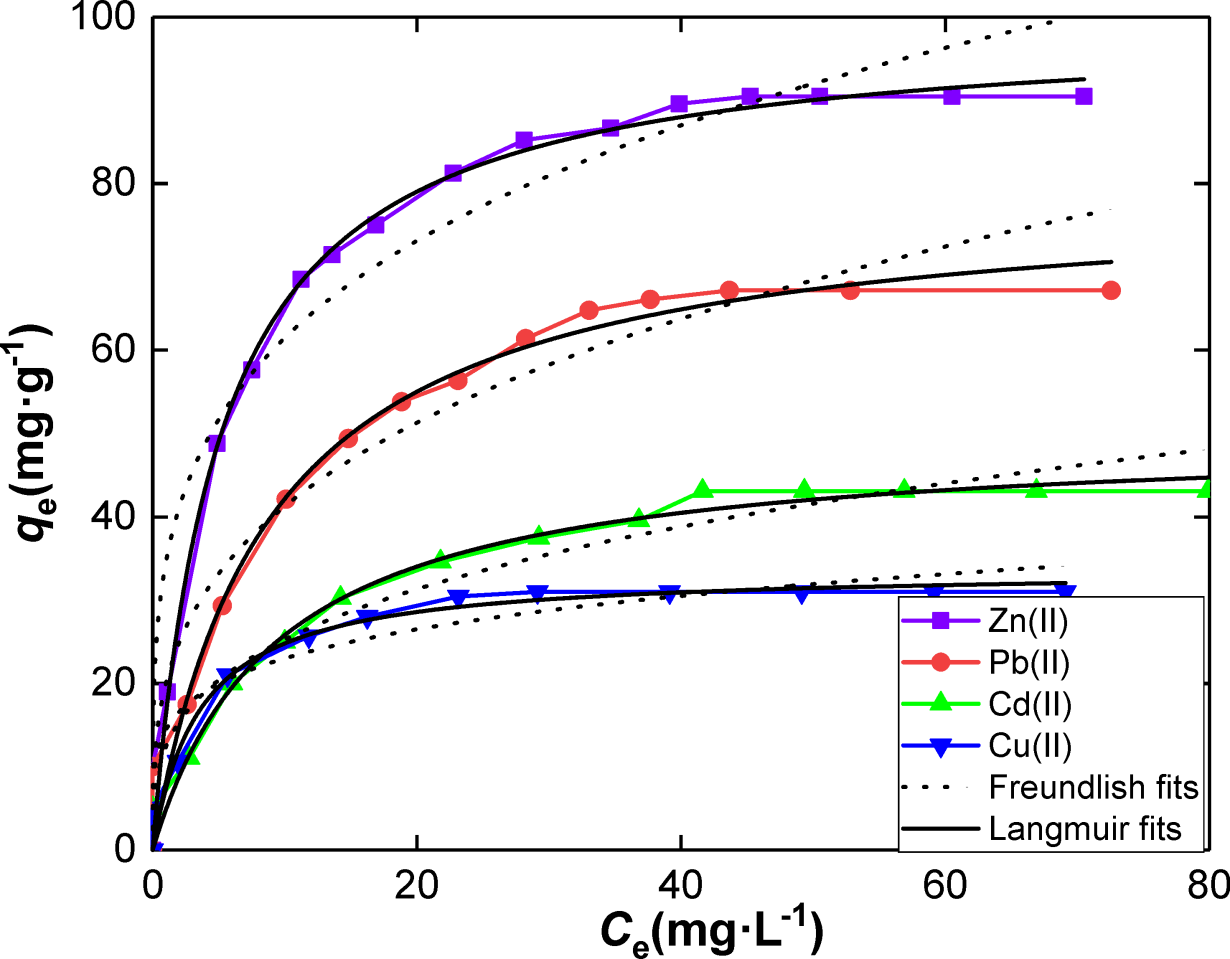
Langmuir and Freundlich adsorption models fits of Zn(II), Pb(II), Cd(II) and Cu(II)) on SiNL.

The adsorption parameters resulting from both isotherm models applied in this work to a selection of toxic metal ions on SiNL are listed in Table 2. Clearly, the best fits were obtained with the Langmuir model (Figure 9). This indicates that the adsorption of metal ions occurs by a monolayer formation in the same limited number of adsorption sites on a homogeneous adsorbent surface.

**Table 2:** Adsorption isotherm parameters of heavy metals onto SiNL.

Metal	Langmuir isotherm model			Freundlich isotherm model		
	*q* (mg·g^−1^)	*K*_L_ (L·mg^−1^)	R^2^	*K*_F_ (mg·g^−1^)	*N*	R^2^

Zn(II)	99.23 ± 1.77	0.19 ± 0.01	0.991	34.53 ± 3.61	3.99 ± 0.48	0.949
Pb(II)	79.11 ± 2.88	0.11 ± 0.01	0.982	20.00 ± 2.96	3.18 ± 0.42	0.943
Cd(II)	49.94 ± 1.37	0.10 ± 0.01	0.988	12.39 ± 1.66	3.23 ± 0.38	0.957
Cu(II)	33.70 ± 1.05	0.278 ± 0.05	0.978	14.41 ± 2.12	4.92 ± 1.02	0.929

**Thermodynamic modeling.** The influence of temperature on the adsorption of Zn(II), Pb(II), Cd(II) and Cu(II) ions onto SiNL was evaluated too. The adsorption thermodynamic parameters (Table 3) were calculated with the van 't Hoff equation [67] which is recalled below:

[7] $$K_{\text{d}}=\frac{C_{0}-C_{\text{e}}}{C_{\text{e}}}\frac{V}{m}\;,$$

**Figure 10:**
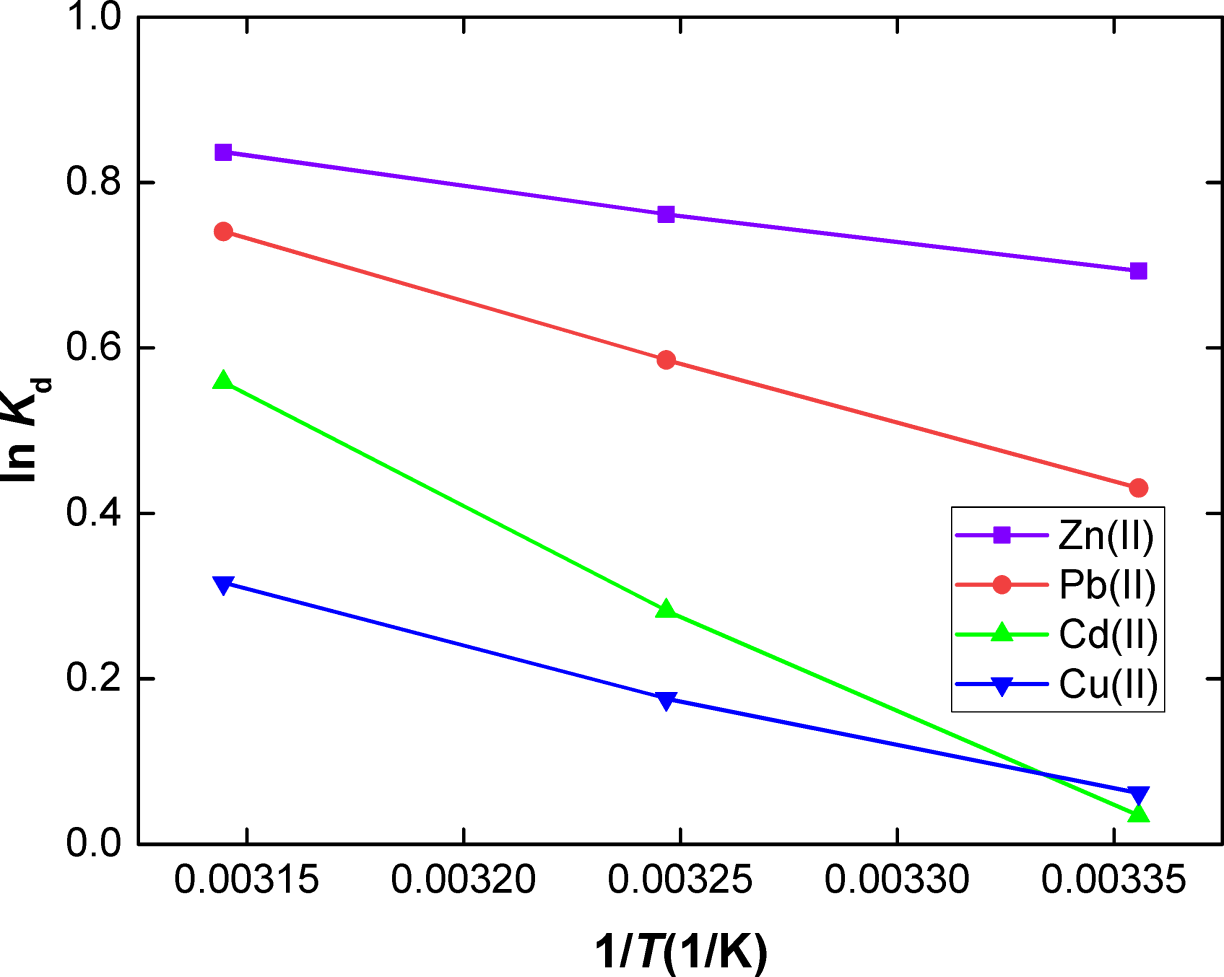
Effect of temperature for the sorption of metal ions onto SiNL (shaking time 60 min, pH 6, adsorption dose: *V* = 10 mL, *m* = 10 mg of SiNL at optimum concentration: 100 ppm of each metal).

[8] $$\ln K_{\text{d}}=\frac{\Delta S^{0}}{R}-\frac{\Delta H^{0}}{RT}\;,$$

where *C*_0_ (mg/L) is the initial concentration of metal solution, *C*_e_ (mg/L) is the equilibrium concentration, *V* (mL) is the volume of solution and *m* (g) is the dosage of sorbents. The Δ*H*^0^ and ΔS^0^ values were derived from the slop and intercept of ln *K*_d_ vs 1/*T* as shown in Figure 10.

**Table 3:** Thermodynamical parameters.

Metal	Δ*H*^0^ (kJ·mol^−1^)	Δ*S*^0^ (J·K^−1^·mol^−1^)	*T* (K)	Δ*G*^0^ (kJ·mol^−1^)

Zn(II)	0.68	24.78	299.15	−06.72
			309.15	−06.97
			319.15	−07.22
Pb(II)	1.48	44.74	299.15	−11.90
			309.15	−12.35
			319.15	−12.79
Cd(II)	2.50	69.76	299.15	−18.36
			309.15	−19.06
			319.15	−19.76
Cu(II)	1.21	34.18	299.15	−09.01
			309.15	−09.34
			319.15	−09.69

A value of ∆*G*^0^ < 0 at all temperatures indicates a spontaneous reaction whereas ∆*H*^0^ > 0 indicates an exothermic adsorption process, which is more favored at low temperatures. ∆*S*^0^ > 0 suggests a higher randomness at the solid solution interface during the adsorption of Zn(II), Pb(II), Cd(II) and Cu(II) onto SiNL.

**Figure 11:**
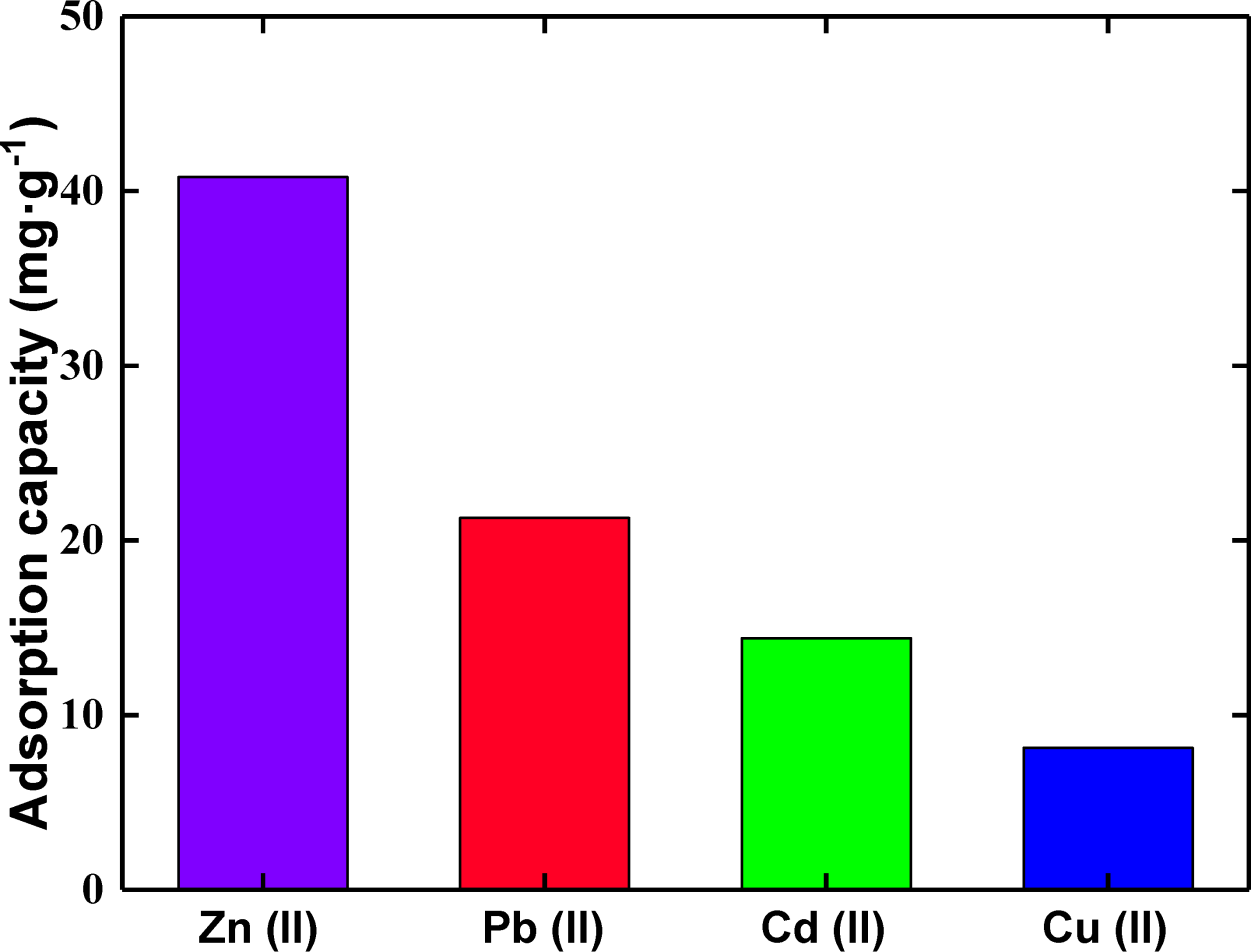
Effect of metal ions on the extraction of Zn(II) with SiNL. (shaking time: 25 min, pH 6, *T* = 25 °C. Adsorption dose: *V* = 10 mL, *m* = 10 mg of SiNL at optimum concentrations: 100 ppm of each studied metal, Pb(II), Cu(II), Zn(II), and Cd(II).

**Selectivity of SiNL.** A remarkable selectivity of SiNL towards Zn(II) (at optimum conditions) compared to other metal ions is shown in Figure 11. The adsorption capacity of SiNL to the metal ions studied is in the order Zn(II) > Pb(II) > Cd(II) > Cu(II). This result is interesting because of the negative influence of zinc on rivers. For example, in Norway, zinc has been found in salmon [68] at concentrations that can kill fish and alter their physiology [69]. Since salmon is a common dish consumed by humans, body damage from consumption can be expected, including cerebral, prostatic, respiratory and gastric abnormalities [70]. Soil contaminated with zinc is also well documented [71]. Many factors can be thought to explain the observed selectivity of SiNL, such as the properties of the metal ions (size, charge, nature), the properties of the grafted ligand (its concentration, its chelating force), and also the properties of the material (its specific surface, nature of the pores). It is therefore quite normal to obtain different adsorption affinities of the same material towards different metals.

**Stability and reusability of the adsorbent.** Our hybrid material can be reused more than five times without significant loss in adsorption efficiency (Table 4). This can be explained by the high stability of organic groups onto SiNL which was confirmed by TGA, showing no distinct changes in the sorbent material after five cycles of use. This suggests that SiNL has excellent chemical stability as a highly efficient adsorbent for the recovery of Zn(II), Pb(II), Cd(II) and Cu(II) ions.

**Table 4:** Adsorption/regeneration of hybrid material towards Zn(II).

Cycle number	Zn(II) (mg·g^−1^)

1	90.48
2	90.16
3	90.06
4	89.76
5	89.12

**Extraction of heavy metal in natural real water samples.** The mesoporous SiNL adsorbent was also used for in-field metal ion removal. Two samples were selected from Morocco rivers: (i) Ghiss (Al Hoceima), (ii) Touissit-bou-bekker (Jerada-Oujda). All samples were collected with a polyethylene bottle and used without storage. The river water was filtered through a 0.45 μm nylon membrane. The ability of SiNL for the sorption of heavy metal was studied by using the batch method by a mixture of 10 mg of adsorbent with 10 mL of river water and 0.5 mL of 1% HNO_3_ at room temperature for 60 min.

The removal efficiency was investigated under optimal conditions. As shown in Table 5, Zn(II) and Cd(II) were successfully removed from environmental water samples using this adsorbent. Of particular interest, is the Touissit River near Oujda, which crosses a mining site of the oriental region of Morocco. This site is well known to be polluted by As, Zn, Pb, Cu and Cd [72]. As a matter of fact, this heavily polluted water was better purified by our hybrid solid (Table 5) compared to Ghiss water.

**Table 5:** Extraction of heavy metal in natural real water samples.

Water samples	Metal ion	*C*_found_ (mg·L^−1^)	Adsorption capacity (mg·g^−1^)

Ghiss river (Al Hoceima-Morocco)	Zn(II)	1.15	0.43
	Cd(II)	1.45	0.52
	Cu(II)	not detectable	–
	Pb(II)	not detectable	–
Touissit-Boubeker river (Jerada-Morocco)	Zn(II)	12.05	6.89
	Cd(II)	2.25	0.53
	Cu(II)	not detectable	–
	Pb(II)	not detectable	–

**Comparison with alternative materials.** Table 6 shows the adsorption efficiency of SiNL towards Zn(II), Pb(II), Cd(II) and Cu(II), compared to literature results. Considering the adsorbed mass quantity, the adsorption capacity values and the affinity for effective adsorption of metal cations under study, our material shows better performance.

**Table 6:** Comparison of adsorption capacity of SiNL with selected reported sorbents.

Support: silica gel/ligand	Ref.	Metal ion (mg·g^−1^)			
		Zn(II)	Pb(II)	Cd(II)	Cu(II)

this work	–	90.48	67.18	43.10	30.91
bipyrazole	[58]	86.51	35.26	26.96	20.24
gallic acid	[73]	–	12.63	6.09	15.38
1,2,4-triazol-2-ylaminopropyl	[74]	09.15	–	–	13.34
*C*,*N*-pyridylpyrazole	[75]	0.0	9.5	1.4	1.8
resacetophenone	[76]	12.49	13.79	06.49	11.80
acid red 88	[77]	0.79	03.35	01.31	0.76
dithizone	[78]	02.32	08.28	03.93	06.07
1,8-dihydroxyanthraquinone	[79–80]	11.79	15.83	07.89	14.39

## Conclusion

A novel hybrid material based on a silica surface covalently anchored to a new highly chelating β-ketoenol–pyridine–furan receptor was prepared. Interestingly, the best adsorption properties were identified for Zn(II), presumably due to the higher stability of the formed Zn–ligand complex compared to other complexes formed with other metal ions. The SiNL adsorbent could remove Zn(II) (90.48 mg·g^−1^) and, to a lesser extent, Pb(II) (67.18 mg·g^−1^) and Cd(II) (43.10 mg·g^−1^) ions, in addition to a relatively small amount of Cu(II) (43.10 mg·g^−1^) ions. This material showed the strongest selectivity for Zn(II) (40.8 mg·g^−1^), followed by Pb(II) (21.28 mg·g^−1^), Cd(II) (14.4 mg·g^−1^), and Cu(II) (8.12 mg·g^−1^) at optimum adsorption conditions. The hybrid material has a high tolerance limit in natural water. The regeneration of the material was studied for several cycles of adsorption–desorption and showed very low loss of its extraction capacity (−1.36 mg·g^−1^). All of these results suggest that SiNL, which showed superior performance for Zn(II), is a suitable material for the removal of heavy metals from real aqueous solutions – a topic which bears enormous importance in environmental remediation.
